# A discussion of RNA virus taxonomy based on the 2020 International Committee on Taxonomy of Viruses report

**DOI:** 10.3389/fmicb.2022.960465

**Published:** 2022-10-14

**Authors:** Wen-Guang Yuan, Guang-Feng Liu, Ying-Hui Shi, Ke-Ming Xie, Jing-Zhe Jiang, Li-Hong Yuan

**Affiliations:** ^1^Guangdong Province Key Laboratory for Biotechnology Drug Candidates, School of Biosciences and Biopharmaceutics, Guangdong Pharmaceutical University, Guangzhou, Guangdong, China; ^2^Key Laboratory of South China Sea Fishery Resources Exploitation and Utilization, Ministry of Agriculture and Rural Affairs, South China Sea Fisheries Research Institute, Chinese Academy of Fishery Sciences, Guangzhou, Guangdong, China

**Keywords:** virus taxonomy, protein alignment, ICTV, RNA virus, segmented virus, Baltimore classification

## Abstract

RNA viruses have a higher mutation rate than DNA viruses; however, RNA viruses are insufficiently studied outside disease settings. The International Committee on Taxonomy of Viruses (ICTV) is an organization set up by virologists to standardize virus classification. To better understand ICTV taxonomy and the characteristics and rules of different RNA virus families, we analyzed the 3,529 RNA viruses included in the 2020 ICTV report using five widely used metrics: length, host, GC content, number of predicted ORFs, and sequence similarity. The results show that host type has a significant influence on viral genome length and GC content. The genome lengths of virus members within the same genus are quite similar: 98.28% of the genome length differences within any particular genus are less than 20%. The species within those genera containing segmented viruses also have a similar length and number of segments. The number of predicted ORFs in the RNA viral genomes also shows a strong, statistically significant correlation with genome length. We suggest that due to the high mutation rate of RNA virus genomes, current RNA virus classification should mainly rely on protein similarities rather than nucleic acid similarities.

## Introduction

Virus classification is the process of naming viruses and placing them into a taxonomic hierarchy, as are the classification systems used for cellular organisms. On one hand, on the basis of virus host, viruses can be classified into four types, namely, animal viruses, fungi viruses, plant viruses, or bacteriophages (Bhat and Rao, 2020). On the other hand, to describe viruses more accurately, David Baltimore established a virus classification system based on the manner of messenger RNA (mRNA) synthesis—the Baltimore classification system. This system classifies viruses into seven types: double-stranded DNA (dsDNA), single-stranded DNA (ssDNA), double-stranded RNA (dsRNA), +strand single-stranded RNA (+ssRNA), −strand single-stranded RNA (−ssRNA), single-stranded RNA viruses with reverse transcriptase (ssRNA-RT), and double-stranded DNA viruses with reverse transcriptase (dsDNA-RT). The Baltimore classes remain an integral part of the conceptual foundation of biology (Koonin et al., 2021).

The International Committee on Taxonomy of Viruses (ICTV) was established in 1966 by virologists to standardize virus classification and naming. This established the first complex and complete virus classification system. In the newest ICTV taxonomy, RNA viruses (except ssRNA-RT) are classified into five major groups based on the phylogenetic tree constructed by Koonin et al. (Wolf et al., 2018). Their results show that dsRNA viruses evolved from +ssRNA viruses on at least two independent occasions, whereas −ssRNA viruses evolved from dsRNA viruses. Furthermore, the last common ancestors of the major branches of +ssRNA viruses only encode the RdRp (RNA-dependent RNA polymerase) and a single jelly-roll capsid protein in common with each other (Wolf et al., 2018).

RNA viruses (except ssRNA-RT) mutate rapidly with a mutation rate that is on average ≅2–3 orders of magnitude higher than DNA viruses. Even ssRNA-RT viruses have a mutation rate that is an order of magnitude higher than DNA viruses (Duffy et al., 2008). RNA virus nucleotide substitution rates are estimated to be roughly six orders of magnitude greater than those of corresponding cellular hosts (Holmes, 2009). These RNA virus characteristics inevitably increase the difficulty of classification. To better understand the most recent ICTV taxonomy and the characteristics and rules of different RNA virus families, we analyzed the 3,529 RNA viruses included in the 2020 ICTV report using five widely used metrics: length, host, GC content, number of predicted ORFs, and sequence similarity. Our review will provide support for analyzing the ICTV taxonomy and understanding the similarities and differences of different virus family members at the genome level.

## Materials and methods

### Downloading and construction of RNA virus database

We set up a localized RNA virus database for the 3,529 RNA viruses included in the 2020 ICTV report. Because of a lack genome data, we eventually downloaded only 2,249 nucleic acid sequences (Supplementary Table S1) from the National Center for Biotechnology Information (NCBI) on December 21, 2020.

### Relationships between viral genome length, GC content, number of ORFs, and host

**Figure 1:**
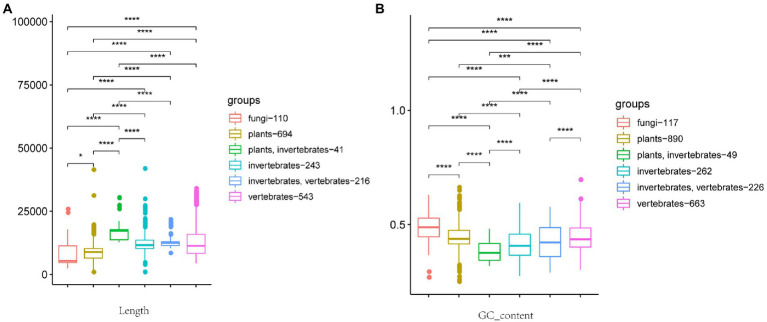
Box plot of relationship between viral genome length, GC content, and host. **(A)** Relationship between viral genome length and host. **(B)** Relationship between GC content and host. The host from left to right is fungi, plants, plants + invertebrates, invertebrates, invertebrates + vertebrates, and vertebrates. The number after the host represents the number of viruses included in the statistics. (Wilcoxon rank-sum test, ^*^p ≤ 0.1, ^****^p ≤ 0.0001.)

GC content and genome length were measured with the “seqkit” package (Wei et al., 2016) available in Linux. The Boxplot was drawn by R. Because the number of viruses infecting some hosts is insignificantly small and/or the complete genome is not available. We only counted those groups with complete genomes and in which the number of viruses infecting a type of host was >40, as illustrated in Figure 1. We annotated the viral genomes with Prodigal (Hyatt et al., 2010) and counted the resulting ORFs.

### Analysis of differences among virus genus levels

To better analyze any possible correlations between the length of a virus genome and the genus level of the virus, we quantified the genus genome differences (equation 1).


(1) $$\mathrm{Genus genome differences}=\frac{\left(\mathrm{Viral genome length}\right)-\left(\mathrm{Average length of inter generic virus genomes}\right)}{\left(\mathrm{Average length of inter generic virus genomes}\right)}$$

### Sequence similarity analyses

The 2,249 RNA virus genome sequences were compared at the protein level using the tblastx function in BLAST (Altschul et al., 1990), and the maximum identity between any two sequences was taken as the protein similarity. Using a custom k-mer algorithm (Kirk et al., 2018) in this study (k-mer set to 10), the 2,249 RNA virus genome sequences were also compared at the nucleic acid level. The nucleic acid similarity between the sequences was calculated by equation (2).


(2) $${\mathrm{Similarity}}_{K-mer}=2^{*}\frac{\left(\mathrm{Number of the same segments}\right)}{\left(\mathrm{Summer of the}\;\mathrm{segments}\right)}$$

## Results

### Adjustment of ICTV To RNA virus taxonomy

The ICTV Executive Committee approved the new taxonomic changes in August 2020 (Gorbalenya et al., 2020). In the new version of the taxonomy, they extended the previously established realm *Riboviria* to almost all RNA viruses and retroviruses (Walker et al., 2020). *Riboviria* is now divided into two kingdoms according to viral replication mode. One, *Orthornavirae*, uses RdRp to replicate. Following Wolf et al.’s phylogenetic tree, this kingdom is divided into five phyla (Wolf et al., 2018; Table 1): *Duplornaviricota*, *Kitrinoviricota*, *Lenarviricota*, *Negarnaviricota*, and *Pisuviricota*. The other *Riboviria* kingdom, *Pararnavirae*, uses RT for reverse transcriptional replication. In this kingdom, because of the low number of viruses found so far, only one phylum, *Artverviricota*, exists and is currently divided into six families (Krupovic et al., 2018): *Retroviridae*, *Metaviridae*, *Caulimoviridae*, *Belpaoviridae*, *Pseudoviridae*, and *Hepadnaviridae*. In summary, ICTV taxonomy is still mainly based on the Baltimore classification system. However, the classification has been adjusted and subdivided according to the phylogenetic tree proposed by Koonin et al. (Wolf et al., 2018).

**Table 1:** the 2020 RNA virus ICTV taxonomy.

Realm	Kingdom	Phylum
*Riboviria* (RNA + dsDNA-RT)	*Orthornavirae* (RDRP)	*Duplornaviricota* (dsRNA)
		*Kitrinoviricota* (+ssRNA)
		*Lenarviricota* (+ssRNA)
		*Negarnaviricota* (-ssRNA)
		*Pisuviricota*
	*Pararnavirae* (RT)	*Artverviricota*

### Variation in genome length between hosts

We tabulated the length of 2,249 RNA virus genomes and associated those lengths with hosts. After preprocessing, six major categories were ultimately differentiated according to host type: fungi, plants, vertebrates, invertebrates, vertebrates + invertebrates, and plants + invertebrates. As shown in Figure 1A, RNA virus genome length generally shows significant differences between hosts. Vertebrate + invertebrate is more concentrated in the 12,000 bp category, indicating that the genome size of such cross-host transmissible viruses is closely related to the host. In addition, the average genome size of viruses associated with animal-associated virus groups (vertebrates, invertebrates, vertebrates + invertebrates, and plants + invertebrates) is significantly larger than that of the other two host types (plants and fungi).

### Variation in GC content between hosts

We also tabulated the GC content of each virus genome sequence and associated that value with hosts. As with genome lengths, GC content generally shows significant differences between hosts (Figure 1B). Correspondingly the average GC content of fungi viruses is significantly higher than that of other groups (Figure 1B; Hettiarachchi and Saitou, 2016). Because viruses need to mobilize the function of host cells to replicate and multiply when a virus infects its host, the GC content of a virus genome reflects the GC content of its host genome.

### Differences in genome length and number of ORFs among different taxa

**Figure. 2:**
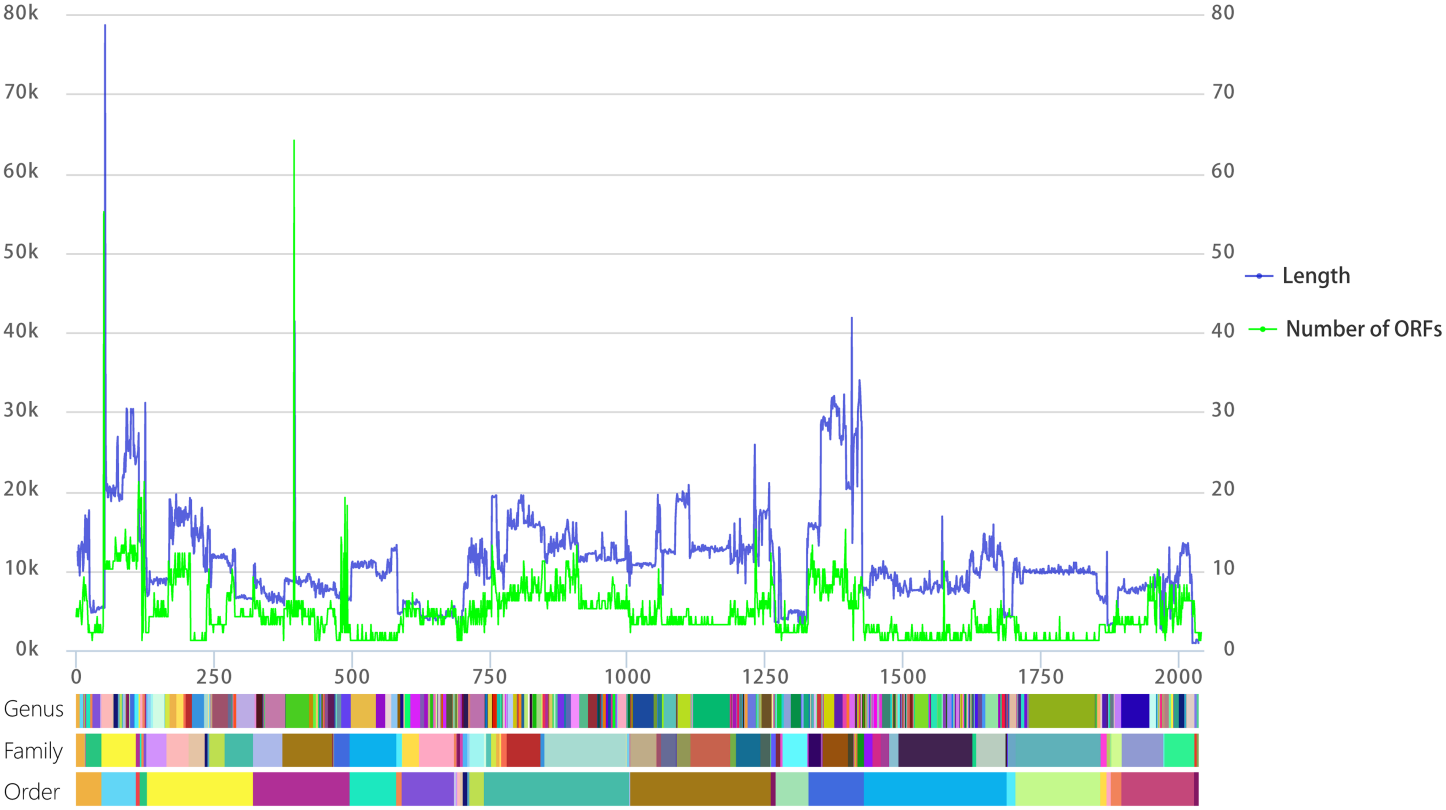
Line chart of viral genome length and the number of ORFs aligned at the level of genus, family, and order. The left vertical axis is the length of the viral genome, the right vertical axis is the number of predicted ORFs, and the horizontal axis represents the cumulative number of viral genomes. The color blocks below correspond to the genus, family, and order from top to bottom, and each different color block represents a different genus, family, or order.

To visualize relationships between virus genome lengths and ICTV virus taxa, we tabulated the 2,035 viral genome lengths of those viruses with complete genomes and aligned those genomes at the order, family, and genus levels. As shown in Figure 2 (blue), viral genome lengths within the same family show good consistency. In particular, the lengths of virus genomes within genera are quite consistent. Except for 35 viral genomes with genus genome differences (equation (1)) are more than 20%, most of the rest (98.28%) of the genus genome differences are less than 20% (Supplementary Table S2)). This means that the length of a viral genome may be used as an important basis for the classification of RNA viruses at the genus level.

In a similar process to our analysis of genome lengths, we visualized relationships between the number of ORFs within the ICTV virus taxa. As shown in Figure 2 (green), the number of ORFs within genera is quite consistent. The number of predicted ORFs in DNA phage genomes shows a strong, statistically significant correlation with genome length (Ha and Denver, 2018). We also analyzed the relationship between the number of predicted ORFs and the lengths of RNA viruses. The number of predicted ORFs in RNA viral genomes again shows a strong, statistically significant correlation with genome length (Supplementary Figure S1).

### Comparison of segmented and non-segmented viruses

**Figure. 3:**
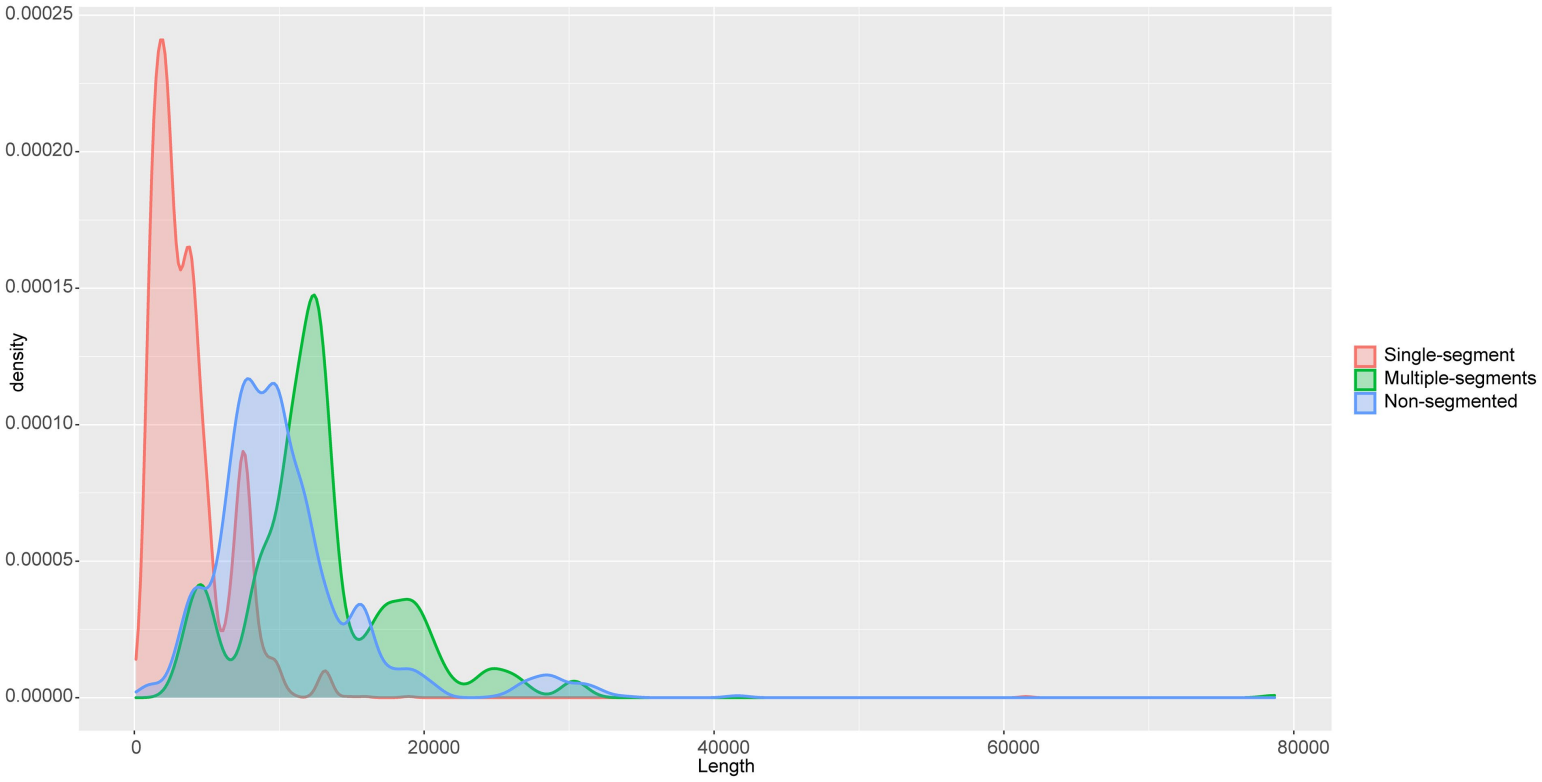
Density plot of genome lengths between single-segment, multiple-segments, and non-segmented viruses. The horizontal axis is the length of the viral genome.

We statistic the genome length of segmented RNA viruses (585 total) in two ways. One is the length of single segment (Single-segment), and the other is the length of total length of all segments of the virus (Multiple-segments). As shown in Figure 3, single-segment (red) viruses have two length peaks, while multiple-segments (blue) and non-segmented (green) viruses have relatively single length peaks. The average length of multiple-segment viral genomes is significantly larger than that of non-segmented viruses (Supplementary Figure S2). Given that long RNA virus genomes are unstable, this suggests that the multiple-segments approach of RNA viruses can better accommodate the instability of RNA genomes. Furthermore, multiple-segments viruses within a genus have similar lengths and numbers of segments (Supplementary Table S3). For example, all viruses in genus *Furovirus* and *Mammarenavirus* are composed of one segment with a length of about 7,500 bp and another around 3,700 bp in length. All the viruses in the family *Bromoviridae* consist of one segment of about 3,500 bp in length and two other segments, each around 2,800 bp in length. It is precisely because of this consistency that the single-segment grouping has two peaks at 3,000 and 8,000 bp (Figure 3).

### Nucleic acid and protein sequence similarity analyses

**Figure. 4:**
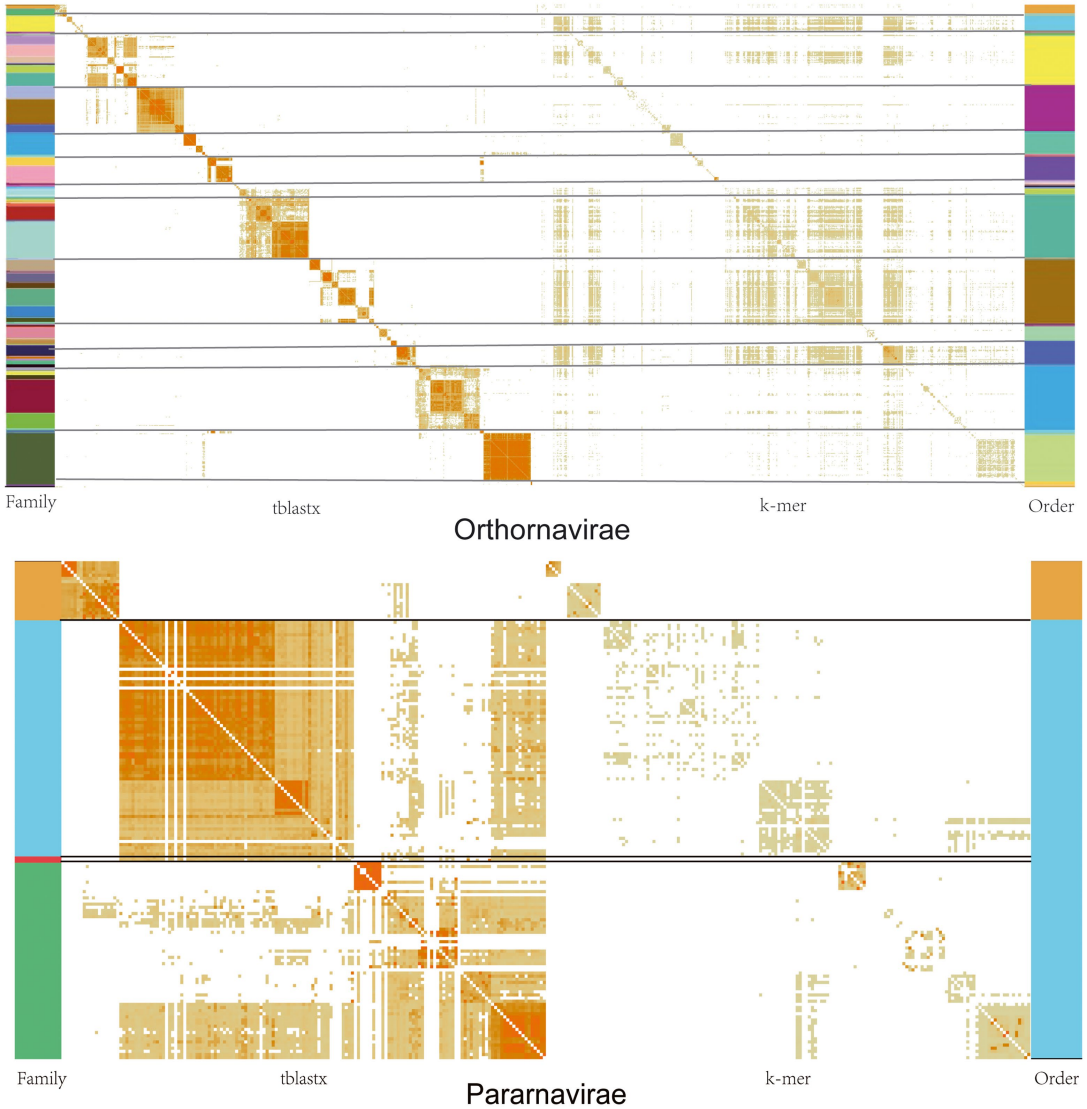
Similarity matrices of viral genomes at the nucleic acid and protein levels in the *Orthornavirae* and *Pararnavirae*. The darker the orange in the figure, the greater the similarity. The different colors on the left represent the classification of different families, and the different colors on the right represent different orders.

We performed pairwise similarity comparisons at the nucleic acid level (k-mer = 10) and protein level (tblastx) for all *Orthornavirae* and *Pararnavirae* genome sequences. As shown in Figure 4, similarity at the protein level in both virus kingdoms shows an obvious clustering effect. This indicates that viruses in the same family or genus have more similar protein sequences, as to be expected. However, similarity at the nucleic acid level does not show an obvious clustering effect. Therefore, due to the high mutation rate of RNA viruses, the classification of RNA viruses should be based on similarity at the protein level.

## Discussion

Holmes described RNA virus evolution as being dominated by mutational processes. Because high error rates place an upper limit on genome size, it is extremely difficult to acquire the additional genetic material needed for a greatly improved polymerase (that is, one possessing some repair function) without suffering a mutational meltdown, as longer genomes result in a greater mutational burden (except in coronaviruses; Holmes, 2009). The results of our study show that the length of an RNA virus genome, whether it is segmented or non-segmented, shows strong regularity. This means that when an unknown RNA virus is taxonomically classified to a known viral family, its genome length should be an important reference factor in its classification.

Furthermore, based on our study, ICTV RNA virus taxonomy should be based on protein similarity rather than nucleic acid similarity. Given the high mutation rate of RNA virus genomes, it is more reasonable to use more conservative protein sequences to classify RNA viruses. This is the reason that most virus classification software, such as vConTACT2 (Bin Jang et al., 2019), CAT (von Meijenfeldt et al., 2019), and PhaGCN2,^1^ are based on protein sequences. Furthermore, even the construction of RNA virus phylogenies should be based on protein sequence alignments. Tools based on nucleic acid sequences, such as Kraken2 (Wood et al., 2019), are limited to the identification of known virus sequences.

Consequently, the ICTV has changed its code to allow a 15-rank classification hierarchy (Gorbalenya et al., 2020). However, the ICTV classification method is still disputed. Although the classification of RNA viruses at the level of family and order is considered valid (RESULT 3.3), it is not enough to classify an RNA virus at the phylum level according to Holmes and Duchene (2019). Presently, it is insufficient to rely solely on phylogeny to reconstruct the evolution of the global virome, but this is no reason to give up on global analyses of virus evolution (Wolf et al., 2019). At present, ICTV relies more on manual comparisons and phylogenetic analyses. However, with the discovery of more and more virus sequences (Wolf et al., 2020; He et al., 2022), current methods are not suitable for a large number of unknown virus classifications (Dutilh et al., 2021). Continuous development and testing of computational tools will be required to maintain a dynamic virus taxonomy that can accommodate new discoveries (Dutilh et al., 2021; Zayed Ahmed et al., 2022). However, the development of new classification tools, such as PhaGCN2 (footnote 1)—a semi-supervised machine learning model to classify viruses based on a graph convolutional network—may be a viable development direction.

## Conclusion

We conducted a statistical analysis of the RNA virus genomes included in the 2020 ICTV report and found that host type has a significant impact on virus genome length, GC content, and segmentation. In particular, virus members within the same genus are more consistent in terms of genome length. Genomes length can be used as an important basis for RNA virus classification. This study also proposes that due to the high mutation rate of RNA virus genomes, the classification of RNA viruses should mainly rely on protein similarity rather than nucleic acid similarity.

## Data availability statement

The original contributions presented in the study are included in the article/supplementary material, further inquiries can be directed to the corresponding authors.

## Author contributions

W-GY: methodology, validation, formal analysis, data curation, writing–original draft, and visualization. G-FL, Y-HS, and K-MX: resources, data curation, and investigation. J-ZJ: conceptualization, methodology, writing–original draft, writing–review and editing, supervision, project administration, and funding acquisition. L-HY: conceptualization, supervision, project administration, and funding acquisition. All authors contributed to the article and approved the submitted version.

## Funding

This project was supported by the Natural Science Foundation of China (nos. 31872499 and 31972847) to L-HY and J-ZJ; the Key-Area Research and Development Program of Guangdong Province (no.2022B1111030001) to J-ZJ; the Central Public-Interest Scientific Institution Basal Research Fund, CAFS (nos. 2020TD42 and 2021SD05) to J-ZJ; and the Guangdong Provincial Special Fund for Modern Agriculture Industry Technology Innovation Teams (no. 2019KJ141) to J-ZJ.

## Conflict of interest

The authors declare that the research was conducted in the absence of any commercial or financial relationships that could be construed as a potential conflict of interest.

## Publisher’s note

All claims expressed in this article are solely those of the authors and do not necessarily represent those of their affiliated organizations, or those of the publisher, the editors and the reviewers. Any product that may be evaluated in this article, or claim that may be made by its manufacturer, is not guaranteed or endorsed by the publisher.

## Footnotes

^1^ https://github.com/KennthShang/PhaGCN2.0
