# Supplementary material for: A discussion of RNA virus taxonomy based on the 2020 International Committee on Taxonomy of Viruses report
Source: Front Microbiol. 2022 Oct 14;13:960465. doi: 10.3389/fmicb.2022.960465 (PMC9615923; doi:10.3389/fmicb.2022.960465)
Supplement: Supplementary file 1 [file Image_1.pdf]

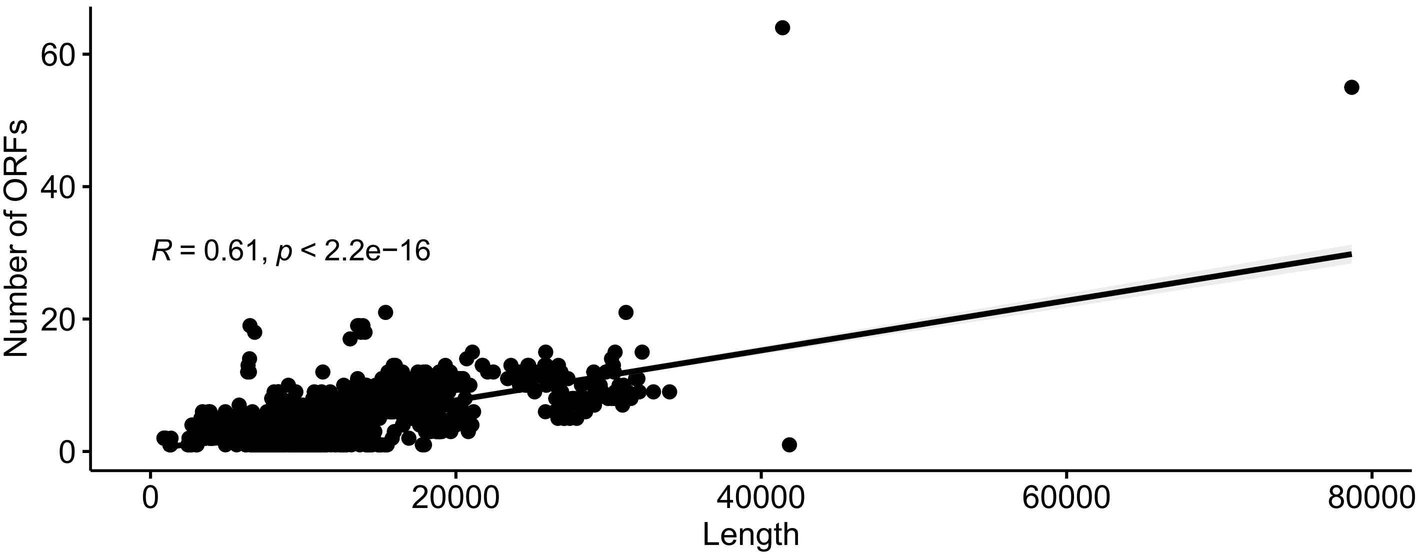

Fig. S1. Scatter diagram of the number of predicted ORFs and the length of viral genomes. The vertical axis is the number of predicted ORFs, and the horizontal axis is the length of the viral genome.
