# Supplementary material for: A discussion of RNA virus taxonomy based on the 2020 International Committee on Taxonomy of Viruses report
Source: Front Microbiol. 2022 Oct 14;13:960465. doi: 10.3389/fmicb.2022.960465 (PMC9615923; doi:10.3389/fmicb.2022.960465)
Supplement: Supplementary file 2 [file Image_2.pdf]

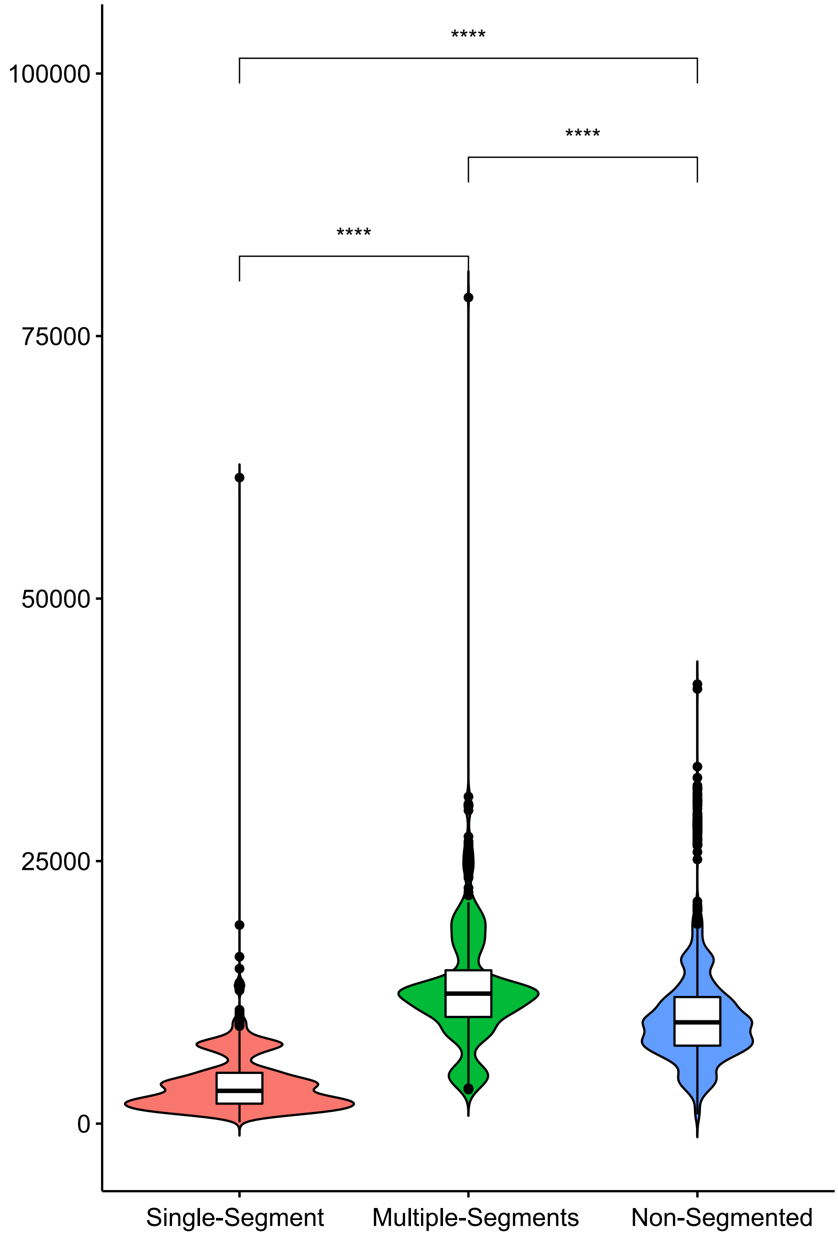

Fig. S2. Violin Plot of the genome length between single-segment, multiple-segments, and non-segmented viruses. The vertical axis is the length of the viral genome. (Wilcoxon rank-sum test, **** $P \leq 0.0001$).
